# A New Benzophenone *C*-Glucoside and Other Constituents of *Pseuduvaria fragrans* and Their α-Glucosidase Inhibitory Activity

**DOI:** 10.3390/molecules23071600

**Published:** 2018-07-02

**Authors:** Wongvarit Panidthananon, Tanawat Chaowasku, Boonchoo Sritularak, Kittisak Likhitwitayawuid

**Affiliations:** 1 Department of Pharmacognosy and Pharmaceutical Botany, Faculty of Pharmaceutical Sciences, Chulalongkorn University, Bangkok 10330, Thailand; naeng_007@hotmail.com (W.P.); Boonchoo.Sr@chula.ac.th (B.S.); 2 Department of Biology, Faculty of Science, Chiang Mai University, 239 Huay Kaew Rd., Chiang Mai 50200, Thailand; tanawat.chaowasku@cmu.ac.th or craibella@hotmail.com; 3 Center of Excellence in Bioresources for Agriculture, Industry, and Medicine, Chiang Mai University, Chiang Mai 50200, Thailand

**Keywords:** benzophenone, glycoside, aporphine, azafluorenone, tyramine amide, α-glucosidase, uncompetitive inhibition

## Abstract

Phytochemical investigations of the leaves and stems of *Pseuduvaria fragrans* led to the isolation of a new benzophenone *C*-glucoside named pseuduvarioside (**1**), together with six known compounds including (−)-guaiol (**2**), (+)-isocorydine (**3**), cyathocaline (**4**), isoursoline (**5**), *N*-*trans*-coumaroyltyramine (**6**), and *N*-*trans*-feruloyltyramine (**7**). Their structures were characterized by NMR spectroscopy and mass spectrometry. All of the isolates were evaluated for inhibitory activity against the enzyme α-glucosidase. *N*-*trans*-coumaroyltyramine and *N*-*trans*-feruloyltyramine showed higher activity than the drug acarbose. Kinetic studies revealed that both tyramine-derived amides were uncompetitive inhibitors of the enzyme.

## 1. Introduction

Diabetes mellitus (DM) is a chronic, metabolic disease typified by unusually high levels of blood glucose. There are several classes of drugs for controlling the blood sugar level. α-Glucosidase is an intestinal enzyme responsible for breaking down carbohydrates into glucose. Inhibitors of this enzyme, for example, acarbose and miglitol, can delay gastric emptying and help to control postprandial hyperglycemia and have been used for the management of DM [1]. A number of phytochemicals have been shown to possess promising α-glucosidase inhibitory activity [2,3]. Our recent study has identified several potent non-competitive inhibitors of this enzyme from *Boesenbergia rotunda* [4].

*Pseuduvaria fragrans* Y. C. F. Su, Chaowasku & R. M. K. Saunders (Annonaceae) was described as a new species in 2010, based on specimens collected from peninsular Thailand [5]. In the Southeast Asian region, about 56 species of the genus *Pseuduvaria* have been recognized, but only a few have been investigated chemically or for a specific bioactivity. Studies on *Pseuduvaria indochinensis*, *P. macrophylla*, *P. monticola*, *P. rugose*, *P. setosa*, and *P. trimera,* revealed prenylated benzopyrans and aporphine alkaloids as their major secondary metabolites [6,7,8,9,10,11,12,13,14,15]. The benzopyran derivatives from *P. indochinensis* and *P. monticola* [6,10], as well as the aporphine alkaloids from *P. rugose*, *P. setosa*, and *P. trimera*, exhibited in vitro cytotoxicity against cancer cells [12,13,14,15]. Recent in vivo experiments on the extracts prepared from *Pseuduvaria macrophylla* and *P. montana* disclosed their antidiabetic effects, which appeared to be mediated by upregulation of insulin secretion [8,11]. Prior to the present study, no reports have appeared on the chemical composition of *Pseuduvaria fragrans*. During our screening of plants for α-glucosidase inhibitory activity, the MeOH extracts prepared from the leaves and stems of *P. fragrans*, at 200 μg/mL, showed 56 and 66% inhibition, respectively and therefore were subjected to further examination. In this communication, we describe our investigation on the constituents of this plant and their inhibitory potential against α-glucosidase.

## 2. Results and Discussion

**Figure 1:**
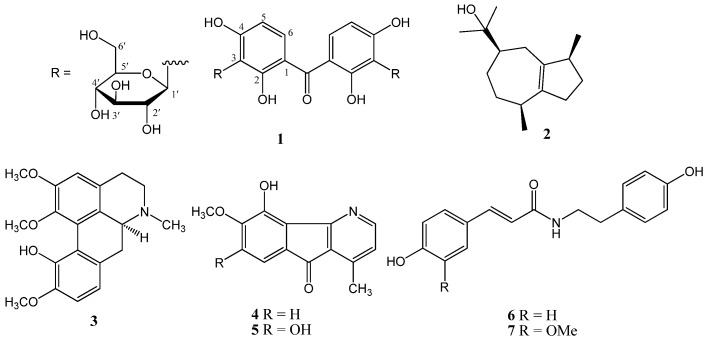
Chemical structures of compounds **1**–**7** isolated from *Pseuduvaria fragrans*.

From the leaves of *Pseuduvaria fragrans*, a new benzophenone glycoside (**1**) was isolated, together with the known compounds (−)-guaiol (**2**) and (+)-isocorydine (**3**), whereas from stems, cyathocaline (**4**), isoursoline (**5**), *N*-*trans*-coumaroyltyramine (**6**), and *N*-*trans*-feruloyltyramine (**7**) were identified (Figure 1).

### 2.1. Structure Characterization

Compound **1** was obtained as a yellow amorphous solid. The HR-ESI mass spectrum showed a sodium-adduct molecular ion [M + Na]^+^ at *m*/*z* 593.1485 (calcd. for C_25_H_30_O_15_Na; 593.1482), suggesting the molecular formula C_25_H_30_O_15_. The UV spectrum of **1** exhibited maximal absorptions at 206, 230, and 275 nm, whereas the IR spectrum displayed bands for carbonyl (1731 cm^−1^) and hydroxyl (3400 cm^−1^) functionalities. The ^13^C NMR spectrum of **1** in CD_3_OD exhibited only 13 signals (Table 1), suggesting that **1** has a symmetrical chemical structure.

The 90° and 135° DEPT spectra indicated a resonance for a carbonyl carbon (δ 198.6), and six aromatic carbon signals attributable to four quaternaries (δ 105.2, 133.9, 160.6, and 162.6) and two methines (δ 115.5 and 132.5). In the aliphatic region, the spectra revealed the presence of six oxygen-bearing carbons, comprising five methines (δ 82.5, 80.0, 77.3, 74.3, and 71.0) and a methylene (δ 62.1). These ^13^C NMR properties were reminiscent of a *C*-glucosidic benzophenone structure [16,17]. From the MS and ^13^C NMR data, as well as the ^1^H–^13^C HSQC correlations, it could be inferred that **1** was a symmetrical benzophenone glucoside containing a glucose unit and two phenolic groups on each aromatic ring. In the ^1^H NMR spectrum, the presence of a pair of *o*-coupled aromatic protons at δ 7.47 (*d*, *J* = 8.1 Hz, H-6) and δ 6.65 (*d*, *J* = 8.1 Hz, H-5) suggested that the three substituents were positioned adjacent to one another and also next to the carbonyl carbon. This was confirmed by the 3-bond HMBC correlation of H-6 with the ketone carbon (δ 198.6). The methine protons of the hexose moiety displayed overlapping and poorly-resolved ^1^H NMR signals, a phenomenon frequently observed for *C*-glycosidic benzophenones [16,17]. When measured in CD_3_OD, the anomeric proton (H-1′) of the glucose moiety appeared to give a resonance at around δ 4.84, as deduced from the HSQC correlation, but the signal was buried under the residual H_2_O peak. In the ^1^H NMR spectrum of **1** recorded in DMSO-*d*_6_, this proton gave a doublet signal (*J* = 9.6 Hz) at δ 4.65, and this indicated a β-configuration for the hexose unit. The 3-bond couplings of H-1′ with the two oxygenated aromatic carbons at δ 160.60 and 162.60 (C-2 and C-4) placed the glucose unit at C-3, and this was corroborated by the ^3^*J* HMBC correlations from H-6 to C-2 and C-4. Thus, **1** was characterized as a new dihydroxybenzophenone diglucoside, and named pseuduvarioside.

The known compounds (**2**–**7**) were identified, through comparison of their spectroscopic and physical properties with literature values, as the sesquiterpene (−)-guaiol (**2**) [18], the aporphine alkaloid (+)-isocorydine (**3**) [19], the azafluorenones cyathocaline (**4**) [20], and isoursoline (**5**) [21] and the cinnamoyl tyramides *N*-*trans*-coumaroyltyramine (**6**) [22] and *N*-*trans*-feruloyltyramine (**7**) [23].

The occurrence of benzophenone derivatives in Annonaceae is indeed rare. So far, they have been reported from only two members of this plant family, i.e., *Cleistochlamys kirkii* and *Polyalthia cerasoides* [24,25]. Tyramine amides also have a rather narrow distribution, being found only in the genera *Annona*, *Enicosanthum*, and *Uvaria* [26,27,28,29]. On the other hand, aporphines and azafluorenones are tyrosine-derived alkaloids known to be widely produced by Annonaceous plants.

### 2.2. α-Glucosidase Inhibitory Activity

Compounds **1**–**7** were subjected to α-glucosidase inhibitory activity evaluation. The cinnamoyl tyramides **6** and **7** showed strong activity (IC_50_ 0.58 ± 0.08 and 3.58 ± 0.13 μM, respectively) with potency higher than that of the drug acarbose (IC_50_ 985.6 ± 35.04 μM). The other compounds **1**–**5** were devoid of activity (<50% inhibition at 100 μg/mL).

**Figure 2:**
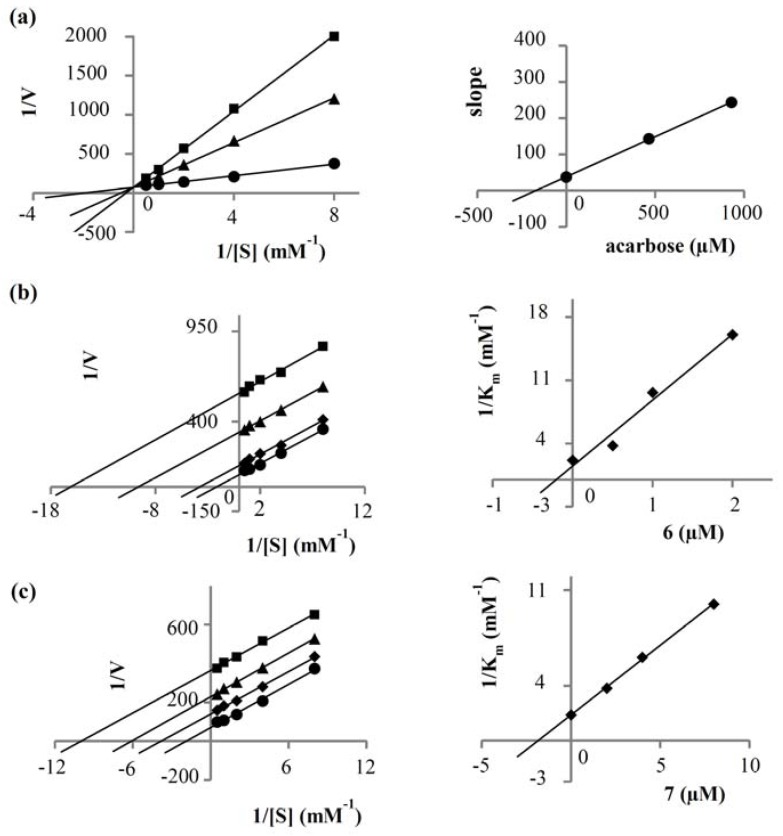
Lineweaver-Burk plots of (**a**) acarbose: ● control, ■ acarbose 600 µg/mL, ▲ acarbose 300 µg/mL; (**b**) *N*-*trans*-coumaroyltyramine (**6**): ● control, ■ (**6**) 2 µM, ▲ (**6**) 1 µM, ◆ (**6**) 0.5 µM; (**c**) *N*-*trans*-feruloyltyramine (**7**): ● control, ■ (**7**) 8 µM, ▲ (**7**) 4 µM ◆ (**7**) 2 µM. The secondary plot of each compound is on the right.

Kinetics studies were then carried out on **6** and **7** to analyze their mode of enzyme inhibition, in comparison with that of acarbose. Lineweaver-Burk plots of the inverted values of velocity (1/V) versus the inverted values of substrate concentration (1/[S]) were prepared. The drug acarbose showed the intersection of the lines on the ordinate, indicative of competitive inhibition. A secondary plot constructed by replotting the slopes of the lines against inhibitor concentration gave a K*_i_* value of 172.27 µM. For amides **6** and **7**, however, parallel lines were obtained in the double reciprocal plots. This was because both the K*_m_* and the V*_max_* values were reduced in equal proportion when the inhibitor concentration increased. These observations suggested that both amides were uncompetitive inhibitors of α-glucosidase. The findings agreed with an earlier report on the uncompetitive α-glucosidase inhibition of **6** and related cinnamic acid amides [30]. To determine the K*_i_* of each amide, we constructed a secondary plot by replotting the reciprocal of K*_m_* (1/K*_m_*) against inhibitor concentration [31]. The intersection on the abscissa yielded a K*_i_* value of 0.20 and 1.83 µM for **6** and **7**, respectively. These kinetic parameters are summarized in Table 2 and Figure 2.

Antidiabetic drugs such as acarbose and miglitol are α-glucosidase competitive inhibitors. Uncompetitive inhibitors of this enzyme have been rarely reported [30]. The amides **6** and **7**, with a unique mode of action, could serve as lead structures for the development of new drugs for controlling after-meal blood glucose levels. As mentioned earlier, the extracts obtained from *Pseuduvaria macrophylla* and *P. montana* could attenuate hyperglycemia in diabetic rats, but their active constituents were not clearly identified [8,11]. In addition, their effects on α-glucosidase have not yet been examined, and this issue may warrant further investigation.

## 3. Materials and Methods

### 3.1. General Experimental Procedures

Vacuum liquid chromatography (VLC) and column chromatography (CC) were performed on silica gel 60 (40–63 μm, Merck, Darmstadt, Germany), silica gel 60 (63–200 μm, Merck, Darmstadt, Germany) or Sephadex LH-20 (Pharmacia, Piscataway, NJ, USA) or Diaion HP20 (Mitsubishi Chemical, Tokyo, Japan). For preparative HPLC, a Shim-pack Prep-ODS (No.2025820) column (Shimadzu, Tokyo, Japan), with isocratic 50% methanol in water, SPD-10A UV-Vis detector (Shimadzu, Tokyo, Japan), and flow rate 1 mL/min, was used. NMR spectra were obtained with a Bruker Avance DPX-300 FT-NMR spectrometer (Brucker Corporation, Billerica, MA, USA). High-resolution electrospray ionization mass spectra (HR-ESI-MS) were recorded with a Bruker micro TOF mass spectrometer (Bruker Daltonics, Billerica, MA, USA). Optical rotations were obtained with a PerkinElmer 341 polarimeter (PerkinElmer, Boston, MA, USA). UV spectra were measured on an Agilent Technologies Cary 60 UV-Vis (Agilent, Santa Clara, CA, USA), and IR spectra (Agilent, Santa Clara, CA, USA) were recorded on a Perkin-Elmer FT-IR 1760x spectrophotometer (PerkinElmer, Boston, MA, USA). Yeast α-glucosidase enzyme, *p*-nitrophenol-α-D-glucopyranoside, and acarbose were purchased from Sigma Chemical, Inc. (St. Louis, MO, USA). Absorbance in 96-well plates was measured using a microplate reader (Wallac1420 Multilevel counter, Victor3, PerkinElmer).

### 3.2. Plant Materials

The leaves and stems of *Pseuduvaria fragrans* Y. C. F. Su, Chaowasku & R. M. K. Saunders were collected from Nopphitam district, Nakhon Si Thammarat, Thailand. Authentication was performed by one of us (T.C.) (Faculty of Science, Chiang Mai University) [1]. A voucher specimen (K. Aongyong 1) has been deposited at the Herbarium Building, Department of Biology, Faculty of Science, Chiang Mai University.

### 3.3. Extraction, Isolation, and Purification

The dried and ground leaves (253 g) and stems (900 g) of *P. fragrans* were each extracted with methanol (5 L) at room temperature for 24 hours for three times. The filtrates were pooled and evaporated under reduced pressure to give a methanol leaf extract (ME-L, 64.9 g) and a methanol stem extract (ME-S, 64.5 g), respectively.

ME-L was suspended in water and partitioned with hexane, EtOAc, and then *n*-butanol to give a hexane extract (10.2 g), an EtOAc extract (11.4 g), an *n*-butanol extract (8.5 g), and an aqueous extract (21.9 g). The hexane extract was separated by VLC on silica gel using a step gradient of hexane-EtOAc to yield 16 fractions (A–P). Fraction I (3.2 g) was further separated by column chromatography (CC) on Diaion HP20 with a gradient mixture of methanol-acetone (1:0 to 0:1) to give five fractions (I1–I5). Fraction I1 (1.1 g) was recrystallized from a mixture of EtOAc-acetone to give white crystals of (−)-guaiol (**2**, 42 mg). The EtOAc extract contained complex and inseparable mixtures of several prenylated benzopyran derivatives, as deduced by NMR analysis. The *n*-butanol extract (8.5 g) was fractionated by CC on Diaion HP20 with a gradient elution (H_2_O, H_2_O-MeOH 3:1, H_2_O-MeOH 1:1, H_2_O-MeOH 1:3, MeOH) to give five fractions (A–E). Fraction B was subjected to CC on silica gel with EtOAc-MeOH polarity gradient elution and further separated by CC (Silica gel, polarity-gradient mixtures of CH_2_Cl_2_-MeOH) to give yellow amorphous solid of a new compound (**1**, 9 mg). Fraction C (1.3 g) was separated on a Sephadex LH-20 (MeOH) column to give 8 fractions (A–H). Fraction C was separated by CC on silica gel (polarity-gradient elution with CH_2_Cl_2_-MeOH) and then purified by preparative RP18 HPLC (Shim-pack Prep-ODS) with isocratic elution (H_2_O-MeOH 1:1; flow rate 1 mL/min) and UV-VIS detection (λ 254 nm) to give a semi-purified alkaloid. Repurification of this residue by preparative RP18 HPLC in a similar manner gave a yellow amorphous solid of (+)-isocorydine (**3**, 11 mg).

ME-S was suspended in water and partitioned with hexane, EtOAc, and then *n*-butanol to give a hexane extract (3.8 g), an EtOAc extract (4.2 g), an *n*-butanol extract (18.5 g), and an aqueous extract (41.0 g). The hexane extract was separated by silica gel CC with polarity-gradient mixtures of hexane-EtOAc to give 16 fractions. Fraction 14 (140 mg) was dried and recrystallized from a mixture of hexane-EtOAc to give a yellow crystalline solid of cyathocaline (**4**, 9 mg). Fraction 15 (509 mg) was separated on Sephadex LH-20 (MeOH) to give nine fractions. Fraction 7 from this column (19 mg) was subjected to CC with hexane-EtOAc polarity-gradient elution to give a yellow crystalline solid of isoursoline (**5**, 14 mg). The EtOAc extract was fractionated by silica gel CC (polarity gradient mixtures of hexane-EtOAc) to give 12 fractions. Separation of fraction 9 (321 mg) by repeated CC, including Sephadex LH-20 (MeOH), silica gel (hexane-acetone polarity gradient), and silica gel (hexane-EtOAc polarity gradient), gave *N*-*trans*-feruloyltyramine (**6**, 8 mg) and *N*-*trans*-coumaroyltyramine (**7**, 4 mg).

Pseuduvarioside (**1**): Yellow amorphous solid; ${\left[\alpha \right]}_{D}^{25}$ + 22.33° (*c* 0.10; MeOH); UV (MeOH) λ_max_ (log ε) 203 (4.19), 230 (3.94), 275 (3.78) nm; FT-IR ν_max_ 3400 (C-OH), 2955 (C-H), 1731 (C = O) cm^−1^; ^1^H and ^13^C NMR data see Table 1; HR-ESI-MS *m*/*z* 593.1485 [M + Na]^+^ (calcd for C_25_H_30_O_15_Na; 593.1482).

(−)-Guaiol (**2**): White crystals; ${\left[\alpha \right]}_{D}^{25}$ − 20.33° (*c* 0.10; MeOH); ^1^H and ^13^C NMR data were identical with reported values [14]; HR-ESI-MS *m*/*z* 245.1886 [M + Na]^+^ (calcd for C_15_H_26_ONa; 245.1881).

(+)-Isocorydine (**3**): Yellow amorphous solid; ${\left[\alpha \right]}_{D}^{25}$ + 139.58° (*c* 0.10; MeOH); UV (MeOH) λ_max_ (log ε) 204 (4.20), 230 (4.25), 275 (3.62), 320 (3.55) nm; ^1^H and ^13^C NMR data agreed with literature values [15]; HR-ESI-MS *m*/*z* 342.1702 [M + H]^+^ (calcd for C_20_H_24_NO_4_; 342.1705).

Cyathocaline (**4**): Yellow crystalline solid; UV (MeOH) λ_max_ (log ε) 210 (4.04), 260 (4.33), 310 (4.01), 355 (3.33); ^1^H and ^13^C NMR data were identical with reported values [16]; HR-ESI-MS *m*/*z* 258.0754 [M + H]^+^ (calcd for C_14_H_12_NO_4_; 258.0766).

Isoursoline (**5**): Yellow crystalline solid; UV (MeOH) λ_max_ (log ε) 205 (4.11), 250 (4.38), 290 (3.84), 305 (3.82), 370 (3.58); ^1^H and ^13^C NMR data agreed with reported values [17]; HR-ESI-MS *m*/*z* 242.0824 [M + H]^+^ (calcd for C_14_H_12_NO_4_; 242.0817).

*N*-*trans*-Coumaroyltyramine (**6**): Yellow amorphous solid; UV (MeOH) λ_max_ (log ε) 225 (4.34), 290 (4.39), 310 (4.38); ^1^H and ^13^C NMR data were in agreement with reported values [18]; HR-ESI-MS *m*/*z* 306.1112 [M + Na]^+^ (calcd for C_17_H_17_NO_3_Na; 306.1106).

*N*-*trans*-Feruloyltyramine (**7**): Yellow amorphous solid; UV (MeOH) λ_max_ (log ε) 220 (4.25), 295 (4.10), 315 (4.13); ^1^H and ^13^C NMR data were superimposable with literature values [19]; HR-ESI-MS *m*/*z* 336.1211 [M + Na]^+^ (calcd for C_18_H_20_NO_4_Na; 336.1212).

### 3.4. Assays for α-Glucosidase Inhibitory Activity

The α-glucosidase enzyme inhibitory activity was assayed by monitoring the release of *p*-nitrophenol from *p*-nitrophenyl-α-d-glucopyranoside (*p*NPG) in 0.1 M phosphate buffer (pH 6.8) [4]. Each test sample was initially dissolved in 50% DMSO. Briefly, 10 µL of the test sample was mixed in a 96-well plate with 40 µL of the α-glucosidase (0.1 unit/mL) in 0.1 M phosphate buffer (pH 6.8) and pre-incubated at 37 °C for 10 min. Then, 50 µL of 2 mM *p*NPG as a substrate was added to start the reaction, and the mixture was incubated at 37 °C for 20 min. The reaction was stopped by adding 100 µL of 0.1 mM Na_2_CO_3_. The absorbance was then measured at 405 nm. 5% DMSO was used as a negative control. Acarbose was used as a positive control and treated under the same condition as the samples. Each experiment was performed in triplicate. Data were displayed as mean ± SD.

### 3.5. Kinetic Study of α-Glucosidase Inhibition

The kinetic assay of enzyme inhibition was done in a 96-well plate. The enzyme kinetics parameters (K*_m_* and V*_max_*) were obtained by analyzing the double reciprocal Lineweaver-Burk plot (1/V vs. 1/[S]). Each experiment was carried out by varying the concentration of *p*NPG (2.0, 1.0, 0.5, 0.25, 0.125 mM) in the absence and presence of different concentrations of the test sample. The reaction was monitored at 405 nm by a microplate reader every 5 min for a total time of 30 min. Each experiment was performed in triplicate. 5% DMSO and acarbose served as the negative and positive control, respectively. A secondary plot for acarbose was generated by plotting the slopes of the double-reciprocal lines versus inhibitor concentration [4]. For compounds **6** and **7**, secondary plots were plots of the inverted values of K*_m_* (1/K*_m_*) as a function of inhibitor concentration [31]. The inhibition constant (K*_i_*) of each compound was then calculated from the intersection on the abscissa.

## 4. Conclusions

In this investigation, we isolated a new benzophenone *C*-glucoside named pseuduvarioside (**1**) and six known compounds including (+)-isocorydine (**2**), (−)-guaiol (**3**), cyathocaline (**4**), isoursoline (**5**), *N*-*trans*-coumaroyltyramine (**6**), and *N*-*trans*-feruloyltyramine (**7**) from *Pseuduvaria fragrans*. We found that amides **6** and **7** were stronger α-glucosidase inhibitors than the drug acarbose. The kinetic parameters (K*_m_* and V*_max_*) of **6** and **7** indicated that both were uncompetitive inhibitors of the enzyme. This study is the first report on the chemical constituents and biological activity of *Pseuduvaria fragrans.*

## Figures and Tables

**Table 1:** NMR (CD_3_OD) data of compound **1** (δ in ppm, *J* in Hz).

Position	^1^H *	^13^C *
1	-	133.9
2	-	160.6
3	-	105.2
4	-	162.6
5	6.65 (d, 8.1)	115.5
6	7.47 (d, 8.1)	132.5
1′	4.84 **	77.3
2′	3.70 (m)	74.3
3′	3.38 (m)	80.0
4′	3.38 (m)	71.0
5′	3.30 (m)	82.5
6′	3.68 (m)	62.1
Carbonyl	-	198.6

* Solvent signal as reference. ** Overlapped with signal of residual water.

**Table 2:** Kinetic parameters of α-glucosidase inhibition in the presence of **6** and **7**.

Inhibitors	Dose (µM)	Slope	V*_max_* ΔA_405_/min	K*_m_* (mM)	K*_i_* (µM)
None	-	37.15	0.0144	0.53	-
*N*-*trans*-coumaroyltyramine (**6**)	2.0	35.56	0.0018	0.06	0.20
	1.0	34.67	0.0030	0.10	
	0.5	34.55	0.0077	0.27	
*N*-*trans*-feruloyltyramine (**7**)	8.0	36.51	0.0027	0.10	1.83
	4.0	37.56	0.0044	0.16	
	2.0	36.62	0.0072	0.26	
